# Building a Standardized Cancer Synoptic Report With Semantic and Syntactic Interoperability: Development Study Using SNOMED CT and Fast Healthcare Interoperability Resources (FHIR)

**DOI:** 10.2196/76870

**Published:** 2025-09-25

**Authors:** Jieun Hwang, Alexander K Goel, Brian A Rous, George Birdsong, Paul A Seegers, Stefan Dubois, Thomas Rüdiger, Walter S Campbell

**Affiliations:** 1 Department of Pathology and Microbiology, University of Nebraska Medical Center, 42nd and Emile, Omaha, NE, 68198, United States, 1 402-559-9593; 2 Topology Health, Toronto, ON, Canada; 3 Department of Histopathology, Cambridge University Hospitals NHS Foundation Trust, Cambridge, United Kingdom; 4 Department of Pathology and Laboratory Medicine, Emory University School of Medicine at Grady Hospital, Atlanta, United States; 5 Palga Foundation, Houten, The Netherlands; 6 Department of Pathology, Antwerp University Hospital, Edegem, Belgium; 7 Institute of Pathology, Städtisches Klinikum Karlsruhe, Karlsruhe, Germany

**Keywords:** semantic interoperability, syntactic interoperability, synoptic pathology report, SNOMED clinical terms, fast healthcare interoperability resources, structured data capture

## Abstract

**Background:**

Pathology reports contain critical information necessary for the management of cancer patient care. Efforts to structure pathology cancer reports by the College of American Pathologists and the International Collaboration on Cancer Reporting (ICCR) have been successful in standardizing pathology reports. Likewise, standards development organizations have advanced methods to improve data computability and exchange, by enabling interoperability of pathology cancer reports.

**Objective:**

This study aimed to provide a tractable method to render pathology cancer reports computable and interoperable using published cancer reporting protocols, SNOMED Clinical Terms (SNOMED CT) and Health Level 7 (HL7) Fast Healthcare Interoperability Resources (FHIR).

**Methods:**

The ICCR colorectal cancer (CRC) reporting dataset (version 1.0) was evaluated by terminologists and pathologists. SNOMED CT concepts were bound to the data elements. The dataset was then converted into a FHIR structured data capture (SDC) questionnaire using the United States National Library of Medicine tooling and rendered into a FHIR-conformant message for data exchange.

**Results:**

The ICCR CRC dataset contained 216 data elements; 207 data elements were bound to SNOMED CT and incorporated into a FHIR SDC construct. The 9 uncoded data elements were ambiguous and could not be reliably encoded. The resultant FHIR SDC form fully represented the ICCR CRC dataset and rendered these data in an R4 JSON format for data exchange.

**Conclusions:**

This study demonstrates a tractable and extensible approach to making cancer pathology reports fully computable and interoperable that can be broadly adopted. ICCR datasets are supported internationally and supported by multiple national pathology societies. These datasets can be fully represented using SNOMED CT to render data elements computable and semantically faithful to their intended meaning. The use of the FHIR SDC construct enables widespread and standardized data exchange of clinical information. While challenges remain, including FHIR adoption and the need to maintain current clinical content and standard terminology, this approach provides a clear pathway toward implementation.

## Introduction

Pathology assessment of tissue specimens is the standard for cancer diagnosis and prognosis, and structured synoptic reporting for cancer pathology reports has been shown to improve report quality [1-8], ease of use [9,10], and patient outcomes [8,11]. However, rendering pathology cancer synoptic reports as computable, electronically interoperable, and exchangeable has been elusive [12]. Developments in computable terminologies and data exchange methods, specifically SNOMED Clinical Terms (SNOMED CT) and Fast Healthcare Interoperability Resources (FHIR) [13,14], provide a pathway toward achieving computable and interoperable pathology cancer reports. This paper demonstrates the use of SNOMED CT and FHIR to produce a fully computable, electronic pathology cancer report for international use.

### Cancer Synoptic Reporting

Multiple national and international bodies of pathology, including the College of American Pathologists (CAP) [15], the International Collaboration on Cancer Reporting (ICCR) [16], the Royal College of Pathologists (RCPath) [17], and the Dutch Nationwide Pathology Database (Palga) [18], develop and publish cancer pathology reporting protocols based on prevailing medical knowledge. These protocols are referred to as synoptic reporting protocols or datasets and prescribe specific data elements that are to be collected and reported. Data include the primary site of tumor, histologic type, histologic grade, anatomic structures invaded directly or metastatically by the tumor, lymph nodes involvement, and, in some protocols, prognostic or predictive immunohistochemistry stains. Protocol guidelines define core and noncore information for inclusion in the report. Core data elements are essential for the cancer’s clinical management, staging, or prognosis for the patient, and for the development of an appropriate treatment plan. Noncore elements represent desirable information that may improve patient care based on emerging science. The benefits of synoptic cancer pathology reporting are abundant, and this reporting approach has been broadly deployed [19]. However, the benefits of structured synoptic reports are not fully realized as the information is not structured in a computable and readily exchangeable format.

**Figure 1.**
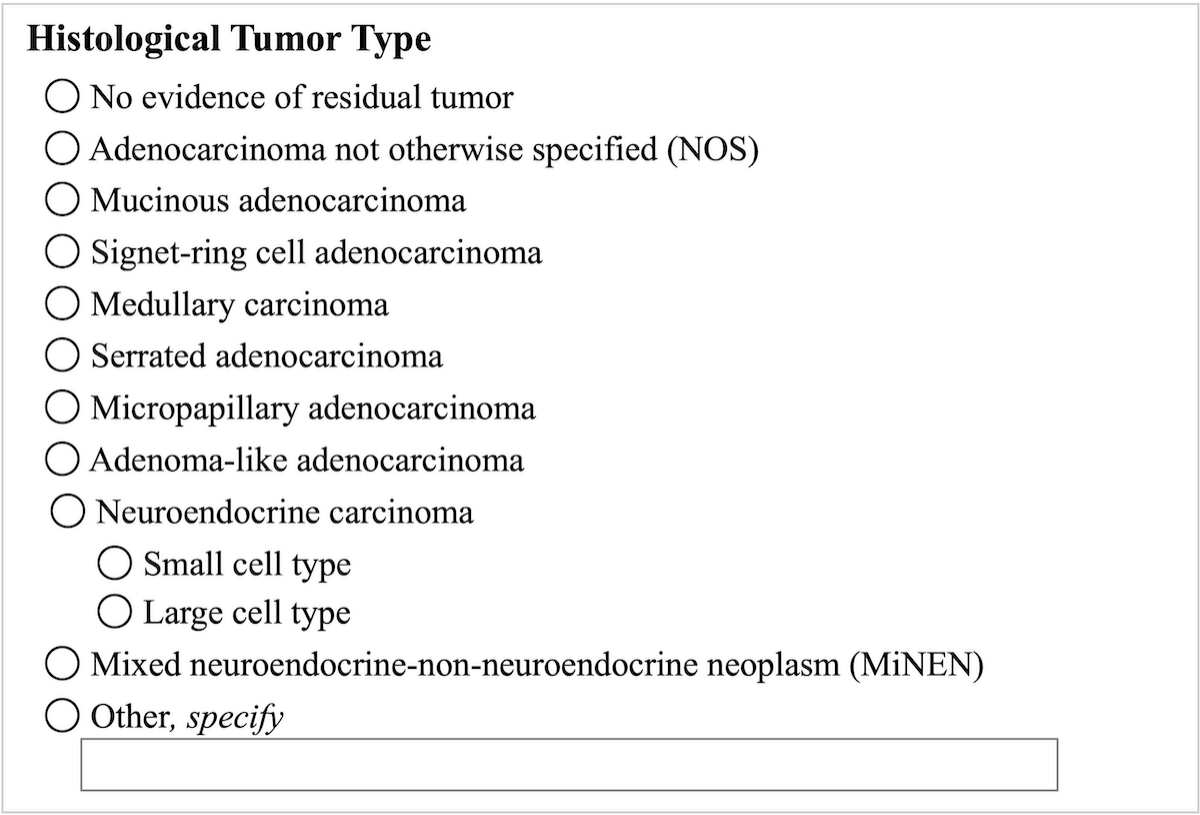
Synoptic pathology reporting template: –histologic tumor type of colorectal cancer.

In practice, pathology cancer reporting protocols are structured into a series of categories to be assessed by the pathologist. Each category contains a limited number of predetermined assessments or observations. For example, the pathologist is required to assess and report the histologic type of the neoplasm. As shown in Figure 1 for colorectal cancer (CRC), the histologic type of the neoplasm should be selected from the constrained list of the most frequent CRC histologic types. A fill-in option is available for rare morphologies.

### Interoperability

At the most basic level, synoptic reports are complex “check sheets” on which pathologists select the appropriate observation for each category (see Figure 1). In a digital environment, check sheets are rendered graphically. Upon completion of the graphical user interface (GUI), corresponding text is created, stored, and often distributed as a PDF or other rich text format. While users easily understand the text, it is not represented with computable terminologies. Thus, the report is reduced to a textual document and not readily available for computation, secondary use, or electronic data exchange. The evolution of pathology cancer reporting from free-text reports to structured reports ensures that pathology reports are easily understood by users and can be computable using controlled medical terminology [8,20]. Recent developments in SNOMED CT content specific for use in pathology cancer reporting provide the avenue to achieve the goal of semantic, computable representation of pathology cancer reports [21].

In addition to standardized terminologies, interoperability of pathology cancer data requires an information model and a data exchange structure. The cancer synoptic reporting formats provided by the CAP, ICCR, RCPath, and Palga ensure a common information model to which standard terminology, specifically SNOMED CT, is bound. FHIR builds on modern web standards technologies to provide meaningful options for data exchange in a variety of health care contexts and provides a scalable data exchange protocol for pathology cancer report data exchange. This review provides a tangible example of using the pathology cancer synoptic structure, SNOMED CT, and FHIR to represent and allow for the exchange of a pathology report. This approach is consistent with international data exchange and interoperability objectives and shows promise for widespread use.

## Methods

### Resource Selection

#### Dataset

The ICCR CRC dataset (version 1.0) [22] was selected to demonstrate the tractability and plausibility of a standardized, interoperable cancer pathology report for broad, international application. The ICCR is a collaboration of pathologists from multiple international professional and academic colleges and societies of pathology [23]. The ICCR publishes pathology cancer reporting guidelines and datasets that are internationally free for use and have been adopted by several nations as the basis for pathology cancer reporting. The colorectal dataset was selected as an example, as this form of cancer occurs across the globe.

#### Terminology

SNOMED CT was used as the base terminology standard for this project. It is widely recognized as the most comprehensive clinical terminology worldwide. The SNOMED CT concept model allows for extensive terminology expression to support explicit concept definition, and the use of description logic enables concept inferences and enhanced data navigation [24]. The recent development of SNOMED content specific to cancer pathology reporting became available in SNOMED CT in late 2023. This content was developed by an international group of pathologists, data scientists, and registrars and was specifically developed for the cancer synoptic reporting use case.

#### Data Structure

In addition to terminology, common information models facilitate data exchange between health care systems by providing structure and syntax for data. FHIR is a data exchange structure with international appeal and serves as a standard for achieving syntactic interoperability in health care [24,25]. Due to the widespread interest in FHIR, this syntactic model was employed in this project, notably the Structure Data Capture (SDC) questionnaire. SDC was developed as part of an initiative sponsored by the US Assistant Secretary for Technology Policy from 2013‐2016 [26]. During this project, the XML version maintained by Integrating the Healthcare Enterprise (IHE) and a FHIR version of SDC were used [27]. The CAP uses the IHE SDC to represent its cancer checklists. The United States National Library of Medicine form builder tool (version 9.7.2) [28] was used to develop an FHIR-compliant cancer synoptic report questionnaire.

### Interoperable Cancer Reporting Template Development

#### Evaluation of Data Elements and Structure

To develop an SDC of the ICCR CRC dataset, each category of information to be assessed by the pathologist and set of potential observations (ie, constrained value set) was evaluated.

**Figure 2.**
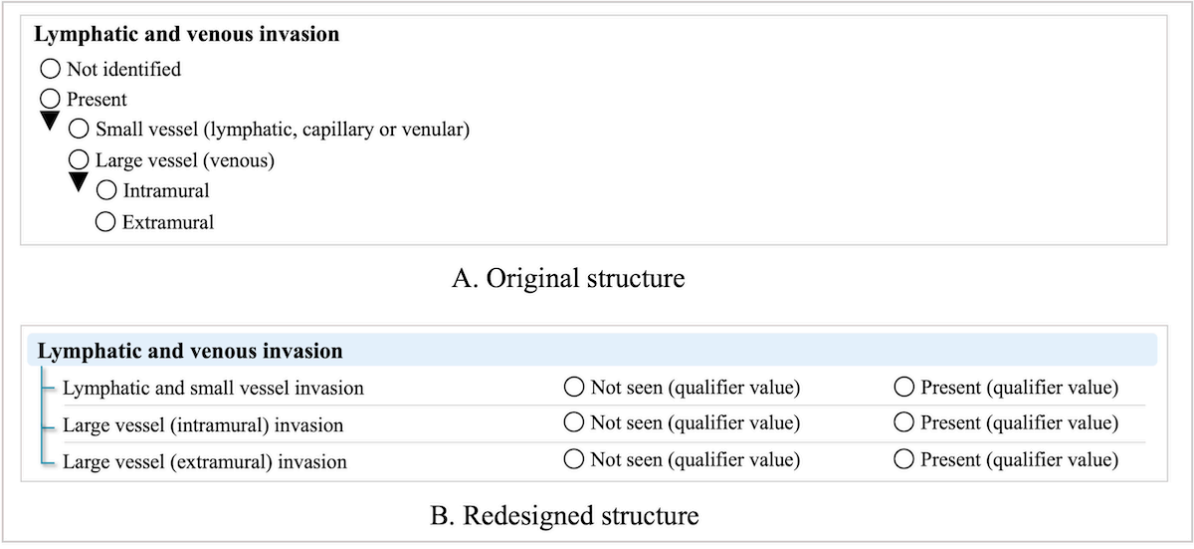
Reconstruction (separate question): lymphatic and venous invasion.

Categories of observations are often “nested” or grouped into logical subcategories when represented in reporting protocols. Such categories can be semantically different, and reporting of subcategories can be contingent upon assessments of predicate subcategories. This condition requires the ability to selectively (ie, logically) solicit observations based on prerequisite information and to associate the semantically correct SNOMED CT concepts for each subcategory of observations. For example, the overarching category of lymphovascular invasion nests three separate semantics into a single category. The observation to be made is the presence or absence of direct invasion in particular lymphovascular spaces: specifically small vessels (ie, venule, arteriole, or lymph vessel), large extramural blood vessel (ie, vessels in pericolic tissue), or large intramural vessels (ie, vessels in the colonic submucosa or colonic muscularis propria). To accommodate these categories and accurately represent each category’s semantic meaning, the overarching lymphovascular category was reconstructed into separate questions (ie, subcategories; see Figure 2).

**Figure 3.**
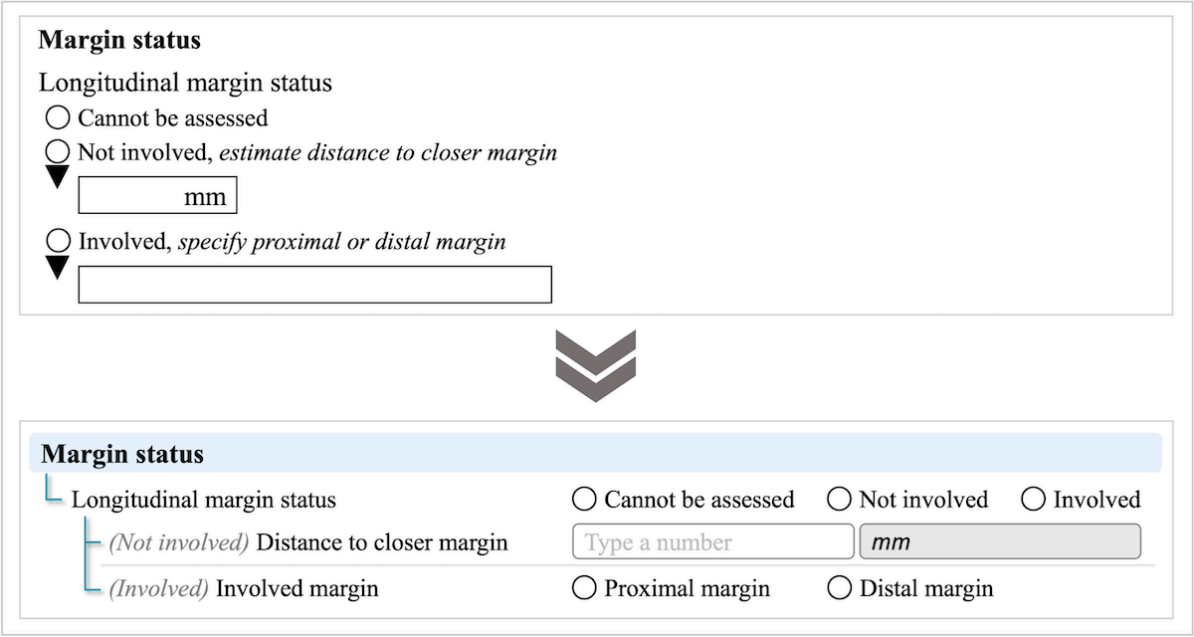
Reconstruction (data type convert): margin status.

The ICCR datasets and those of the CAP are published with observation value sets that consist of the list of expected or plausible assessments. These lists do not represent all possible assessments, which would be unwieldy, if not impossible, to represent in a dataset. Therefore, a free-text option is made available for most categories of assessments. Nevertheless, where possible, we converted free-text fields into structured data entry. For example, we changed the data type from free text to an answer list for assessments of involved surgical margins based on ICCR supporting notes. Free-text data types require human intervention, consume significant time, and are prone to error. Automatically identifying the specific data within free-text fields is not feasible. To minimize human intervention, we specified which margin was involved by carcinoma, as shown in Figure 3. In the original CRC dataset, pathologists were required to describe the surgical margin if the longitudinal margin was involved by carcinoma. We changed this data element into two answers: proximal margin and distal margin.

#### Terminology Binding

After reconstruction, SNOMED CT concepts from the latest version of SNOMED International (version 2025-02-01) were associated (ie, bound) with each data element. Following the synoptic style, (ie. question and answer pair structure), the context for any recorded observation is represented in the observable entity concept; we referred to it as the observable entity approach. Table 1 shows an example of SNOMED CT terminology using this observable entity approach for histologic type of CRC malignant neoplasm.

**Table 1.** Example of terminology binding - Histological tumor type.

Element value	SNOMED ID and fully specified name
Question	
Histological tumor type	1284862009 |Histologic type of primary malignant neoplasm of cecum and/or colon and/or rectum (observable entity)|
Answer	
No evidence of residual tumor	41647002 |No evidence of (contextual qualifier) (qualifier value)|
Adenocarcinoma	1187332001 |Adenocarcinoma (morphologic abnormality)|
Mucinous adenocarcinoma	72495009 |Mucinous adenocarcinoma (morphologic abnormality)|
Signet-ring cell adenocarcinoma	87737001 |Signet ring cell carcinoma (morphologic abnormality)|
Medullary carcinoma	32913002 |Medullary carcinoma (morphologic abnormality)|
Serrated adenocarcinoma	450948005 |Serrated adenocarcinoma (morphologic abnormality)|
Micropapillary adenocarcinoma	450895005 |Micropapillary carcinoma (morphologic abnormality)|
Adenoma-like adenocarcinoma	28558000 |Villous adenocarcinoma (morphologic abnormality)|
Neuroendocrine carcinoma	1286767006 |Neuroendocrine carcinoma (morphologic abnormality)|
Small cell type	74364000 |Small cell carcinoma (morphologic abnormality)|
Large cell type	128628002 |Large cell neuroendocrine carcinoma (morphologic abnormality)|
Mixed neuroendocrine-non-neuroendocrine neoplasm (MiNEN)	785766008 |Mixed neuroendocrine-non neuroendocrine neoplasm (morphologic abnormality)|
Other	74964007 |Other (qualifier value)|
Other, specify	This response is not codable data.

#### Building an SDC

**Figure 4.**
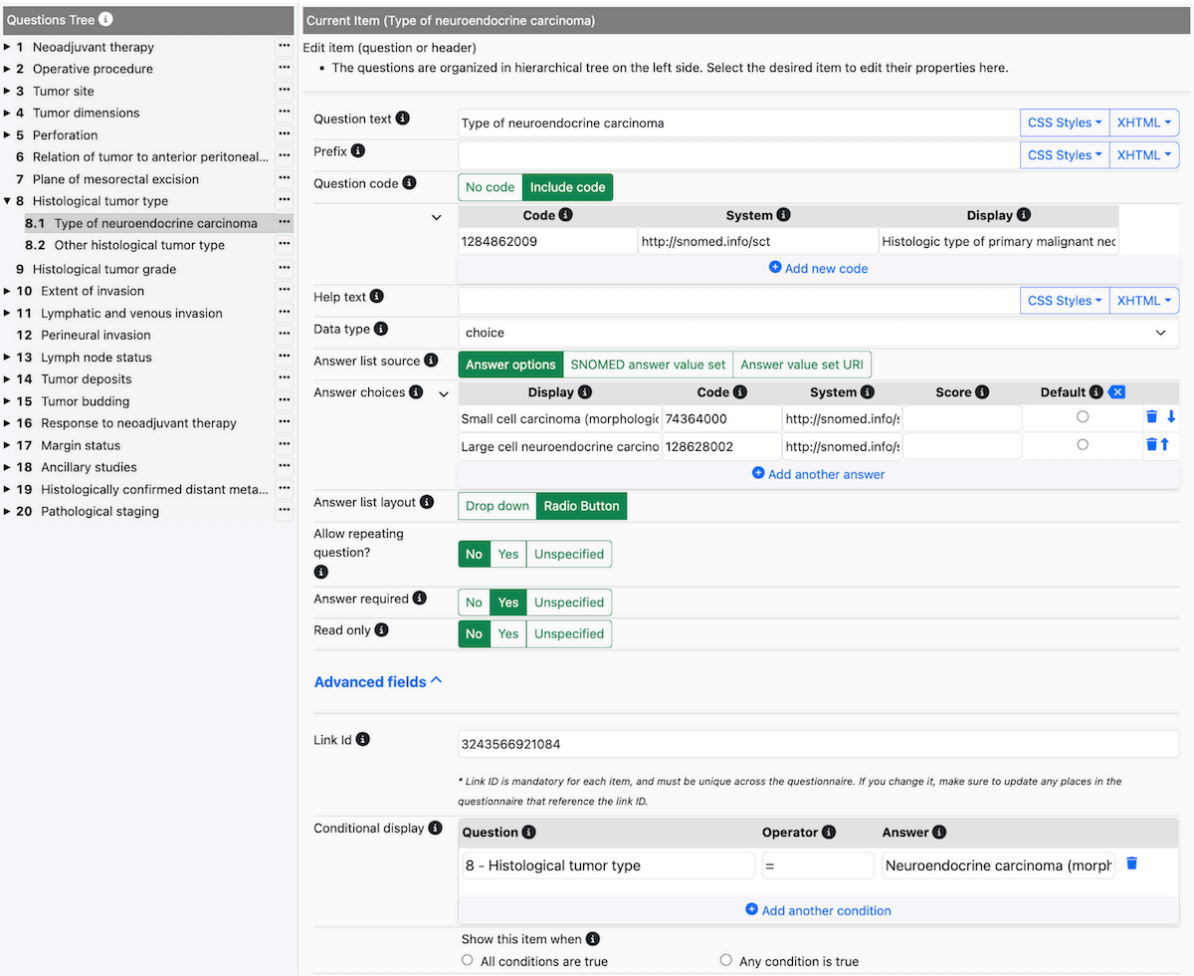
The National Library of Medicine (NLM) form builder (version 9.7.2).

The NLM form builder tool was then used to represent the category for assessment and the value sets of responses in an FHIR-compatible SDC (see Figure 4). Natural language consistent with the ICCR template was used for the user interface (ie, question text). At the same time, the data layer and semantic meaning of the recorded observations made in the SDC were represented using a reference to SNOMED CT. Most data elements were qualitative values, but several assessments to be made by the pathologist required discrete or numerical entries. In these situations, the NLM SDC data element was configured to solicit and accept a numerical entry. These included categories of assessments pertinent to tumor dimensions (size) and numbers of lymph nodes examined and involved.

### Ethical Considerations

This study did not involve human participants and therefore was not subject to institutional ethical review.

## Results

### Targeted Elements and Reconstruction

The CRC dataset contains a total of 25 categories (ie, items to be assessed by the pathologist), comprising 19 core categories and 6 noncore categories. After evaluating the value of data elements, all data elements from core categories and some noncore categories were selected as target items. Following reconstruction based on meaning, the standardized CRC dataset from ICCR was converted into 45 questions and 171 answers.

### Building Results

Table 2 summarizes the SNOMED CT concepts used for binding. Of the 216 data elements in the ICCR CRC protocol, 207/216 (95.8%) were able to be bound to SNOMED CT concepts. All questions bound with SNOMED CT concepts used the semantic tag (observable entity). For answers, concepts with the semantic tags (qualifier value), (body structure), (morphologic abnormality), (procedure), and (tumor staging) were used accordingly. Answer values are not necessarily unique to the question, and SNOMED CT answer concepts represent answer or observation values across several question categories. For example, ‘52101004 |Present (qualifier value)|’ or ‘47492008 |Not seen (qualifier value)|’ are used in several observation categories. The observable entity concept representing the question or category is unique and carries all necessary context by which to interpret the answer or observation. Thus, the same SNOMED CT concepts may be used for multiple answers and still maintain the semantic integrity of the data. Further details are provided in Multimedia Appendix 1 which includes data elements and the corresponding bounded SNOMED CT concepts.

**Table 2.** Summary of the binding results.

Binding status	Semantic tag	Values, n
Question		
Not bound	—^a^	3
Bound	Observable entity	42
Answer		
Not bound	—^a^	6
Bound	Body structure	15
Bound	Morphologic abnormality	13
Bound	Procedure	12
Bound	Qualifier value	122
Bound	Tumor staging	3

a Not applicable.

Table 3 includes unbound data elements and reasons why they are not bound. Among the 9 unbound elements, 2 data elements were added during the construction of the SDC without specific meaning, serving only as part of the logical structure. Of the remaining 7 unbound items, the first, related to ‘Circumferential margin–Involved by primary tumor or other’ is ambiguous and cannot be encoded accurately. Likewise, reference to ‘Ancillary study for neuroendocrine markers’ is not precisely defined by ICCR and cannot be reliably encoded. Finally, changes by the American Joint Committee on Cancer and Union for International Cancer Control regarding tumor, node, metastasis (TNM) descriptor representation require additions to SNOMED CT content and will be bound once a new concept is authored.

**Table 3.** Unbound data elements with their corresponding reasons and solutions.

Type of data elements		Unbound elements	Comment
Question	Answer		
Tumor dimension	Can be assessed	Answer	This item was added during creating the SDC^a^.
Tumor budding	Can be assessed	Answer	This item was added during creating the SDC.
Circumferential margin–Involved by	By primary tumor	Question and answer	The meaning of the elements is unclear.
	By other		
Ancillary studies for neuroendocrine markers	Not applicable	Question and answer	It is difficult to predict what will be recorded here.
	Neuroendocrine markers		
TNM^b^ descriptor	m–multiple primary tumors	Question	Authoring a new concept is requested.
	r–recurrent		
	y–post-therapy		

a SDC: structured data capture.

b TNM: tumor, node, metastasis.

### FHIR Questionnaire

**Figure 5.**
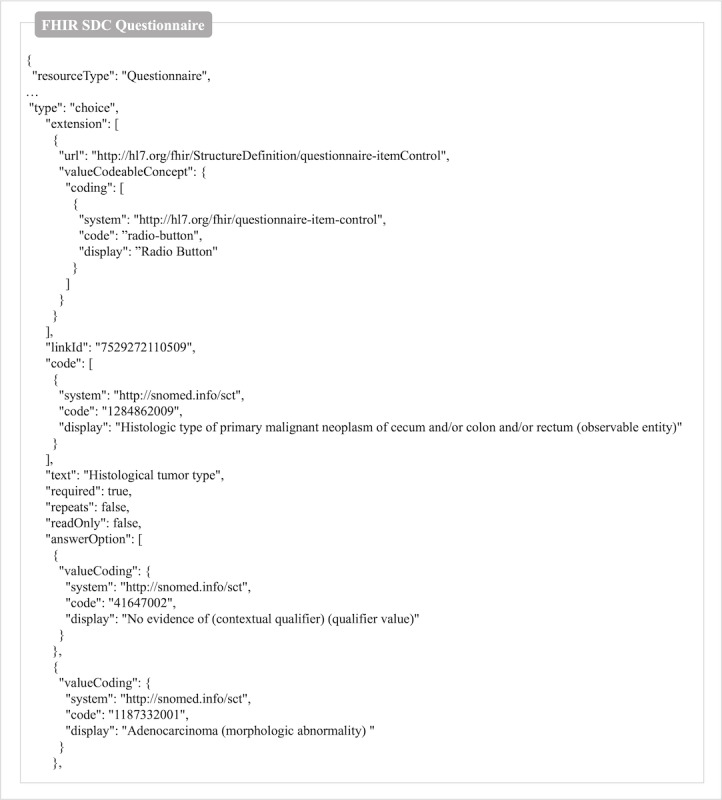
A snapshot of the FHIR SDC questionnaire content in JSON format, R4 version.

Figure 5 is a partial screenshot of the FHIR SDC questionnaire in JSON format. The full version is provided in Multimedia Appendix 2.

## Discussion

### Principal Results

This study describes the creation of a structured CRC ICCR pathology cancer reporting dataset bound with standardized terminology for international use, in a manner compliant with the Health Level 7 (HL7) FHIR standard for syntactic and semantic interoperability. Cancer protocols developed by CAP, ICCR, and other institutions follow a synoptic style. SNOMED terminology development for synoptic use follows this model, and newly authored concepts were used in this study. The observable entity approach is necessary to maintain the encoded question and answer data element pair for data integrity. As a result, the implementation and data storage of synoptic reports must accommodate this pairing. The NLM SDC precisely accommodates synoptic style and retains the question-answer binding, as reflected in Figure 2. Interoperation on a syntactic level, known as syntactic interoperability, is a mainstay of interoperability [29] and facilitates data transmission between information systems. The standards to achieve this are HL7 version 2 (HL7 v2) and FHIR. Electronic synoptic reporting is supported using either version. However, we focused on the FHIR representation to demonstrate the feasibility of using its architecture for pathology reporting, as it leverages open web technologies and represents the future direction of international health data exchange [14].

The primary purpose of the NLM form builder is to create SDC-based questionnaires. The form builder is very user-friendly and supports features such as conditional display, data type and value restrictions, cardinality of responses, valid observation values, and default values. In short, using SDC-based questionnaires facilitates usability while ensuring that well-structured and high-quality data are collected.

Much of this paper addresses data collection, data quality, and data representation. The primary purpose of FHIR is data exchange. HL7 message creation and formatting is a function of the NLM SDC form builder tool. The form builder produces a FHIR Release 3 Standard for Trial Use and Release 4 compliant FHIR message capable of transmission in FHIR format as well as devolution into a compliant HL7 v2 format. Thus, using the approach described in this work, a FHIR-capable method for pathology cancer reporting data exchange is created.

### Challenges

While the findings of this project are promising, it is necessary to consider operational challenges.

#### Version Control

Cancer reporting guidelines evolve over time. In some instances, new SNOMED CT content will be necessary. Therefore, a process for protocol (dataset) version control and terminology binding review must be developed. The ICCR is responsible for maintaining clinical content, and SNOMED International is responsible for terminology content. The organizations have agreed to ongoing collaborations to ensure alignment of terminology with clinical content.

#### Implementation Complexity

Using the ‘observable entity approach’ implemented in cancer synoptic reporting can be more complex than other approaches. In the observable entity approach, the question, or tumor characteristic to be observed, is represented by a SNOMED CT observable entity concept. Observations made and recorded by the pathologist use a variety of SNOMED CT concepts based on the tumor feature benign recorded. The observable entity concept contains full contexts and definitions to unambiguously understand the pathologist’s recorded measurement, which is represented by more generalizable concepts such as histological types, grades, and presence of various other tumor characteristics. As a result, the observable entity concept and corresponding observation concepts must be stored in tandem. This is consistent with other laboratory data types. This approach was deliberate. Existing SNOMED CT concept definitions do not support the development of fully defined clinical finding concepts in which multiple observations are contained within a single concept. Furthermore, developing such an approach would result in an unmanageable, combinatorial explosion of SNOMED CT concepts; for example, colorectal adenocarcinoma, lung adenocarcinoma, endometrium adenocarcinoma, and all possible ‘body site’ x ‘histology type’ combinations. Software developers and clinical information system vendors will need to consider this design feature when developing future applications.

### Future Studies

Although an approach for interoperable synoptic pathology cancer reporting using international standards is presented in this paper, such a system has to our knowledge not yet been implemented. Interoperability, the intended objective of SNOMED CT and FHIR cannot be realized unless the system is fully tested when implemented in the real-world settings. Ultimately, FHIR may offer an improved methodology for storing SNOMED CT coded data and would enhance the exchange and sharing of pathology data. As part of implementation, there will be opportunities to test how FHIR can improve data storage, analytics, and other secondary data use cases. Future activity in this realm should include using FHIR SDC Data Extraction guidance to convert data collected using the ICCR datasets into more readily usable FHIR resources. When a FHIR Questionnaire, such as those authored as part of the methodology of this paper, is rendered in a GUI, the system produces a FHIR Questionnaire Response. Converting the data from the FHIR Questionnaire Response resource to Observations and other applicable resources would enhance the semantic interoperability and ensure that the data can be queried from FHIR-based systems. Further promotion of the benefits of adopting FHIR and incentives for both technology vendors and healthcare providers would improve adoption, as evidenced by the adoption of the standard incentivized by the 21st Century Cures Act in the United States [19], which encouraged significant FHIR adoption across the country.

### Conclusions

The findings of this paper demonstrate that syntactic and semantic interoperability for cancer synoptic reporting is possible using existing international standards. The current international release of SNOMED CT contains the necessary concepts required for synoptic pathology cancer reporting for all solid tumors. Along with FHIR SDC tooling from the United States NLM, we demonstrate the plausibility of FHIR-based encoded pathology cancer reporting on a global scale.

## Supplementary material

10.2196/76870 Multimedia Appendix 1 Data element with SNOMED CT.

10.2196/76870 Multimedia Appendix 2 Colorectal cancer.R4.

## Abbreviations

CAP: College of American Pathologists
CRC: Colorectal cancer
CT: SNOMED Clinical Terms
FHIR: Fast Healthcare Interoperability Resources
GUI: Graphical User Interface
HL7: Health Level 7
ICCR: International Collaboration on Cancer Reporting
IHE: Integrating the Healthcare Enterprise
NLM: National Library of Medicine
Palga: Dutch Nationwide Pathology Database
RCPath: Royal College of Pathologists
SDC: Structured data capture
TNM: tumor, node, metastasis
